# A156 OUTCOMES FOLLOWING ENDOSCOPIC RESECTION OF GASTRIC NEUROENDOCRINE TUMOURS FROM A TERTIARY-CARE ACADEMIC CENTRE

**DOI:** 10.1093/jcag/gwac036.156

**Published:** 2023-03-07

**Authors:** S Gupta, G Brar, K Zheng, S Hakim, C W Teshima, G R May, C H Law, J Hallet, J D Mosko

**Affiliations:** 1 Medicine, University of Toronto; 2 Laboratory Medicine and Pathobiology, St. Joseph’s Health Centre; 3 The Center for Advanced Therapeutic Endoscopy and Endoscopic Oncology, St. Michael’s Hospital; 4 Surgery, Sunnybrook Health Sciences Centre, Toronto, Canada

## Abstract

**Background:**

Gastric neuroendocrine tumours (G-NET) are rare cancers derived from neuroendocrine cells of the stomach. A steady increase in the incidence of these tumours has been observed. Current treatment and surveillance strategies are guided by various tumour characteristics including size, grade, and depth of invasion. There exists conflicting evidence, however, on the rates of recurrence from positive resection margins following primary endoscopic resection. Thus, it remains uncertain whether complete endoscopic resection (R0) of these indolent tumours is clinically significant and whether follow-up endoscopic or surgical intervention is justified.

**Purpose:**

Our aim is to characterize current management patterns and clinical outcomes in patients undergoing endoscopic resection of G-NETs.

**Method:**

We conducted a retrospective, single-centre cohort study at The Centre for Advanced Therapeutic Endoscopy and Endoscopic Oncology at St. Michael’s Hospital, Toronto, Ontario. Consecutive patients over the age of 18 who underwent endoscopic resection of histologically proven G-NETs between 2011 and 2020 were included. Data on patient, endoscopic, and tumour characteristics were collected through electronic chart review. Descriptive statistics were conducted for data analysis.

**Result(s):**

A total of 155 foregut neuroendocrine tumours were endoscopically resected during the study period, of which 108 were identified as G-NETs. 95.3% were classified as Type I. Mean tumour size was 8.93 ± 5.27 mm. Cap-assisted EMR was performed most frequently (n=51), followed by conventional EMR (n=35). ESD was performed in eight cases. Seven intra-procedural perforations occurred, of which all were closed endoscopically. One patient experienced post-procedural perforation requiring ICU and surgery. Positive resection margins (R1) were found in 25% of cases (n=27), of which 78% were assessed at surveillance endoscopy 1 (SE1). Six patients with R1 margins were referred for surgical evaluation and four were lost to follow-up. 78% of all resected G-NETs were followed at SE1 with a median interval of 196 days (range, 23 to 3373). SE1 recurrence rate at the primary resection site was 14% (n=12), of which two were from routine scar biopsies in the absence of endoscopically identifiable recurrence. All visible recurrences at these sites (n=10) were managed with repeat endoscopic resection. Patient and tumour characteristics in the evaluation of G-NET recurrence are presented in Table I.

**Image:**

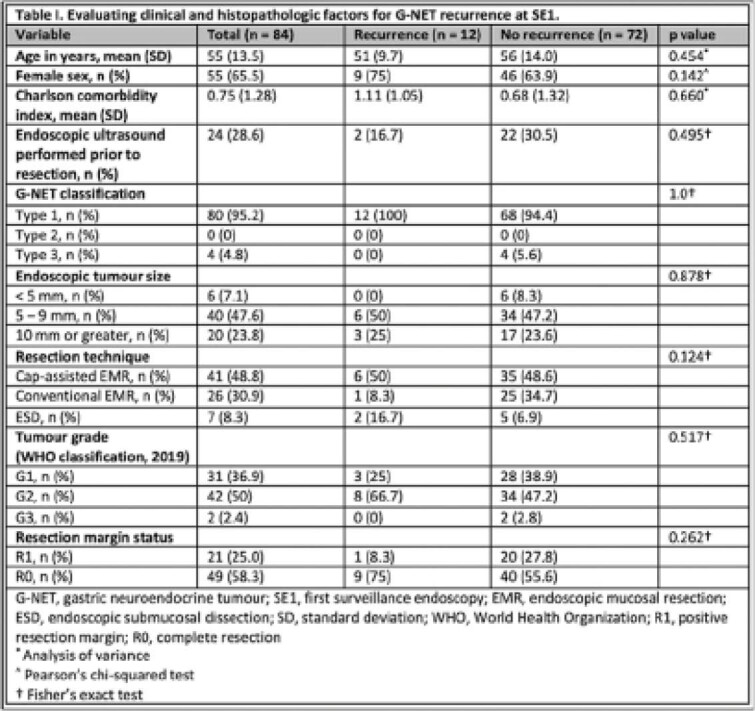

**Conclusion(s):**

G-NET recurrence occurs in less than 15% of patients at surveillance endoscopy following endoscopic resection in spite of a predictably higher R1 resection rate. Patient, endoscopic, and tumour factors including method of resection and margin status do not appear to impact the development of early recurrence. Given the indolent nature of these tumours, patients with positive resection margins can be followed conservatively. Further investigation is warranted to determine the optimal duration and surveillance strategy for these patients.

**Please acknowledge all funding agencies by checking the applicable boxes below:**

None

**Disclosure of Interest:**

None Declared

